# Mutational screening of *EXT1* and *EXT2* genes in Polish patients with hereditary multiple exostoses

**DOI:** 10.1007/s13353-014-0195-z

**Published:** 2014-02-15

**Authors:** Aleksander Jamsheer, Magdalena Socha, Anna Sowińska-Seidler, Kinga Telega, Tomasz Trzeciak, Anna Latos-Bieleńska

**Affiliations:** 1 Department of Medical Genetics, Poznan University of Medical Sciences, Rokietnicka 8 Street, 60-806 Poznan, Poland; 2 NZOZ Center for Medical Genetics GENESIS, Grudzieniec 4 Street, 60-601 Poznan, Poland; 3 Department of Orthopedics and Traumatology, Poznan University of Medical Sciences, 28 Czerwca 1956 r. 135/147 Street, 61-545 Poznan, Poland

**Keywords:** EXT1, EXT2, Hereditary multiple exostoses, Multiple osteochondromas, Mutation, Polish patients

## Abstract

Hereditary multiple exostoses (HME) also known as multiple osteochondromas represent one of the most frequent bone tumor disorder in humans. Its clinical presentation is characterized by the presence of multiple benign cartilage-capped tumors located most commonly in the juxta-epiphyseal portions of long bones. HME are usually inherited in autosomal dominant manner, however de novo mutations can also occur. In most patients, the disease is caused by alterations in the *EXT1* and *EXT2* genes. In this study we investigated 33 unrelated Polish probands with the clinical and radiological diagnosis of HME by means of Sanger sequencing and MLPA for all coding exons of *EXT1* and *EXT2*. We demonstrated *EXT1* and *EXT2* heterozygous mutations in 18 (54.6 %) and ten (30.3 %) probands respectively, which represents a total of 28 (84.9 %) index cases. Sequencing allowed for the detection of causative changes in 26 (78.8 %) probands, whereas MLPA showed intragenic deletions in two (6.1 %) further cases (15 mutations represented novel changes). Our paper is the first report on the results of exhaustive mutational screening of both *EXT1*/*EXT2* genes in Polish patients. The proportion of *EXT1*/*EXT2* mutations in our group was similar to other Caucasian cohorts. However, we found that *EXT1* lesions in Polish patients cluster in exons 1 and 2 (55.6 % of all *EXT1* mutations). This important finding should lead to the optimization of cost-effectiveness rate of HME diagnostic testing. Therefore, the diagnostic algorithm for HME should include *EXT1* sequencing (starting with exons 1–2), followed by *EXT2* sequencing, and MLPA/qPCR for intragenic copy number changes.

## Introduction

Hereditary multiple exostoses (HME) also referred to as multiple osteochondromas (MO) are one of the most common benign bone tumors with an estimated prevalence rate of 1 per 50,000 in European population (Hennekam 1991; Schmale et al. 1994). The disorder is usually inherited in autosomal dominant manner, however de novo mutations are also known to occur. HME are highly penetrant (close to 100 %) and show significant variability in symptoms expression and the age of onset, which varies from 2 to 15 years (Schmale et al. 1994). Clinical picture of HME involves formation of benign cartilage-capped tumors most frequently located in the juxta-epiphyseal parts of long bones, especially around the knee (femur, tibia), the wrist, the proximal humerus, the proximal fibula, and the ribs. Exostoses can also occur in scapula and pelvis, but neither mandible nor the calvarium are involved (Shapiro et al. 1979; Schmale et al. 1994). Lesions are usually inconspicuous at birth and tend to grow in number and size through childhood and adolescence until the closure of growth plates in puberty. It has been suggested that the formation of exostoses in HME patients is the consequence of a two-hit model in which predisposition for tumor development due to germline mutation and a second somatic “hit” are necessary for the development of exostoses (Mertens et al. 1994). Due to the disruption of growth plates osteochondromas may lead to the various skeletal deformities, such as limb shortening, angular deviation of long bones (especially the ulna), Madelung deformity, movement restrictions, as well as short stature. In addition, exostoses can cause nerve or blood vessel compression, joint limitations, and in some cases (up to 5 %) can transform into malignant tumors such as chondrosarcoma or osteosarcoma (Hennekam 1991; Wicklund et al. 1995).

HME result from the mutations of at least two putative tumor suppressor genes, i.e., *EXT1* located on chromosome 8q24.1 and *EXT2*—located on chromosome 11p11 (Ahn et al. 1995; Stickens et al. 1996; Wuyts et al. 1996). Third region with a hypothetical *EXT3* gene was mapped to the chromosome 19p in linkage studies of *EXT1*/*EXT2* negative family, nevertheless causative mutations associated with this locus have not been identified to date (Le Merrer et al. 1994). Mutations in *EXT1* gene underlie 56–78 % of HME cases, whereas in *EXT2*—21–44 % (Jennes et al. 2009). Both *EXT1* and *EXT2* genes encode for glycotransferase enzymes of endoplasmic reticulum (N-Acetylglucosamine transferase and D-glucuronic acid transferase respectively) involved in the synthesis of heparan sulfate and proteoglycans (Busse et al. 2007). Mutations in these genes have different patterns of concentration. While lesions of *EXT1* are scattered throughout the gene, mutations in *EXT2* tend to cluster in the first N-terminal part of the protein (Busse et al. 2007; Jennes et al. 2009).

In this study we investigated 33 unrelated Polish index cases with the clinical and radiological diagnosis of HME. We extend the mutational spectrum of the genes and report our diagnostic experience regarding *EXT1* and *EXT2* screening in Polish patients, who were not represented so far in published molecular studies.

## Materials and methods

### Patients and clinical information

Thirty three unrelated index cases of Polish ethnicity who were clinically and radiographically suspected of HME were recruited for this study. The inclusion criteria involved two or more exostoses diagnosed upon clinical assessment and/or X-ray imaging. Twenty five probands had a positive family history, while eight were sporadic. Blood was collected from all index cases as well as from affected and unaffected available family members. The local ethics committee approved the study and written informed consent was obtained from all subjects or their legal guardians.

### Molecular screening

Genomic DNA was isolated from whole blood according to the conventional salting-out method. The coding sequences of both *EXT1* and *EXT2* genes (GenBank accession number NM_000127 and NM_207122.1), comprised of all coding exons, and the flanking intronic regions were amplified in a set of PCR reactions and directly sequenced by means of dye-terminator chemistry (kit v.3, ABI 3130XL). Sequences of the primers used for amplification and sequencing PCR reactions are given in Table 1 (primers for *EXT1* were as described elsewhere by Baasanjav et al. 2010). Multiplex ligation-dependent probe amplification (MLPA) for all exons of the *EXT1* and *EXT2* was performed with the use of commercial kit P215-B1 per the manufacturer’s protocol (MRC Holland). Data was intra-normalized by dividing the area of each peak by the overall area of the reference probes’ peaks in the probemix. Inter-sample normalization was obtained by comparing the investigated samples to several reference control samples (healthy individuals) run in the same experiment. Relative peak areas ranging from 0.67 to 1.33 were considered normal, below 0.67—deleted, and above 1.33—duplicated (Schouten et al. 2002). DNA of all index cases was screened for both point mutations and intragenic copy number changes involving *EXT1* and *EXT2* gene, by means of sequencing and MLPA. Next, co-segregation testing was performed in all affected and unaffected family members to check for co-occurrence of the detected mutation with the phenotype. Alternatively, parental studies were done in sporadic cases to confirm a de novo occurrence of the alterations. Detected mutations were referred to Human Gene Mutation Database (HGMD) Professional 2013.4 (HGMD) and Leiden Open Variation Database (LOVD) version 2.0 (Fokkema et al. 2011). Pathogenicity of all identified missense variants was additionally assessed in silico using Mutation Taster 2, Polyphen2, and SIFT software.

**Table 1:** Sequences of the primers used for *EXT1* and *EXT2* gene amplification and sequencing

Exon name	F primer sequence 5′-3′	R primer sequence 5′-3′
EXT1 gene		
EXT1_e1a	TCTTTACAGGCGGGAAGATG	TGTTCCACAAGTGGAGACTCTG
EXT1_e1b	CCAGGTTCTACACCTCGGAC	CTCAGTTCCAGGCTCAAAGG
EXT1_e2	CTGGTGGCTTTCCCGAG	AAGGGAAACCACACCTTCTC
EXT1_e3	AAGCTTCCTTTCCTTCTGGC	CCATGACACAGGTAATTTTCTCC
EXT1_e4	TGCTAGAAGCCAAATGCTATG	TGGACCAATCACACATCCC
EXT1_e5	CTCTGACTGCCACCATCTTTC	AAGCAATCTTCAATGCAGGG
EXT1_e6	ATTTGCTCCAGCATGAGGC	TGAATGAAAGGGAGTAGCAGG
EXT1_e7	GCTGAGATTTCCAGCTCCTC	AACAGGGAGAAGATATCTAGGGC
EXT1_e8	AGATTCCTTCGGTGTTGAGG	CAAGGCACGGCTAAAAGAAG
EXT1_e9	CCGGATTTTGCATTATGAATTAG	ATCAGCAAAACTTAAGCGGG
EXT1_e10	GGGATTCAAAGAATGGGTATG	CTGGGTGGAACAGCTAGAGG
EXT1_e11	TGCTCATTTGCCTGACTCC	ACAATCTGGCTCTGCTGATG
EXT2 gene		
EXT2_e2a	CCTGAGTGACAGAGTGAAACCC	GGTTGAAGCCACAGCGATAG
EXT2_e2b	TGATGTGCCGGTTGTTAGG	AGAAGACAGCATCGGGAAAC
EXT2_e3	TTGCATACCTGAGAAGCGG	TCTTCAGGAGGAAAATACTTATGAC
EXT2_e4	CTGACTCTGTAAACGTTAGCTGG	CAGTGCCTCAAGGACCCTAC
EXT2_e5	TCAGTGGAGGTGAAGACTGG	TGCTATGTTTTCTTCCCCTTG
EXT2_e6	GTGAGCTGTTGTCTTTTGGC	GCTCTAGACCAGTGTACTAACTCTCC
EXT2_e7	GTTCAGCCAGTGAAGAAGGG	TTCCTATCGTTTCAGTTTGGC
EXT2_e8	AGCATATGCCCTAGGCACC	AAAAGCACACTCTCATCTTAGAAAG
EXT2_e9	AGCAGTTGCTTAGCTCTGGG	GCATGCTGTCTCAGAAATGG
EXT2_e10	TTTGGATTTGATGAGAGCCG	TCTTACGCACACCTTTTGGAC
EXT2_e11	GGGAGGAAGTCAGAATCAGC	TGGTTATCTCGAAGTGACAGG
EXT2_e12	CATTCTAATGCCTCCTTTTACCC	CAATTTCCCAATGTGACCG
EXT2_e13	GAGTTGAATGGAGGAATGGC	TAACCCAATTCCCACAGTGC
EXT2_e14	GAACCTGGGAGCAGACTGTG	GAAAGTGGGTTAGGTGGGTG

## Results

We found *EXT1* and *EXT2* heterozygous mutations in 28 out 33 (84.9 %) unrelated probands from our cohort. In total, we demonstrated 26 different mutational hits, since two of them were recurrent. Eighteen causative alterations (54.6 %) were identified in *EXT1*, while ten (30.3 %) were shown in *EXT2*. The remaining five cases (15.1 %) were negative for both *EXT1* and *EXT2* mutations. DNA sequencing has allowed for the detection of causative changes in 26 (78.8 %) probands, whereas MLPA showed intragenic copy number changes in two (6.1 %) further cases. Out of 28 molecularly confirmed unrelated probands, six cases (21.4 %) occurred due to de novo mutation while 22 (78.6 %) inherited the disease causing variant from an affected parent. According to HGMD® Professional 2013.4 and LOVD v.2.0 databases, out of 26 different mutations demonstrated in this study, 15 alterations were novel, whereas 11 were previously reported elsewhere (HGMD, Fokkema et al. 2011). Nine out of the 15 novel single nucleotide variants (SNVs) were identified in *EXT1* and six in *EXT2*. For the description of the mutations and their reference to literature data, see Table 2. Out of 28 mutations detected by us, 23 (82.1 %) represented inactivating variants (17 frameshift, three nonsense, three splicing mutations). The remaining causative changes were two intragenic deletions (both in *EXT2*) and three missense substitutions (all in *EXT1*).

**Table 2:** List of mutations in *EXT1* and *EXT2* genes identified in our MO probands

Gene	Exon/intron^a^	Nucleotide change	Protein change	Type of mutation	Case	Reference
EXT1	1	c.15dupA	p.R6Tfs^a^24	frameshift	F	Jennes et al. (2009)
	1	c.214G>T	p.E72^a^	nonsense	F	Wuyts et al. (2005)
	1	c.218delA	p.N73Tfs^a^63	frameshift	S	Jennes et al. (2009)
	1	c.365delA	p.Q122Qfs^a^14	frameshift	F	Novel mutation
	1	c.482delT	p.L161^a^	frameshift	F	Novel mutation
	1	c.698delC	p.S233Lfs^a^19	frameshift	F	Novel mutation
	1	c.812A>G	p.Y271C	missense	F	Fokkema et al. (2011)
	2	c.1019G>A	p.R340H	missense	F	Raskind et al. (1998); Cheung et al. (2001)
	2	c.1036A>G	p.R346G	missense	F	Signori et al. (2007)
	IVS2	c.1056+1G>C	skipping exon 2	splicing	F	Novel mutation
	6	c.1431delC^b^	p.P477Lfs^a^11	frameshift	F^b^	Jennes et al. (2008)
	6	c.1454delA	p.H485Lfs^a^3	frameshift	F	Novel mutation
	6	c.1469delT	p.L490Rfs^a^9	frameshift	F	Ahn et al. (1995); Sarrión et al. (2013)
	9/IVS9	c.1859_1883 + 1dup	p.K628Kfs^a^1	frameshift	S	Novel mutation
	IVS9	c.1883+1G>T	skipping exon 9	splicing	F	Novel mutation
	10	c.1902_1903insTA	p.S635Yfs^a^9	frameshift	S	Novel mutation
	10	c.2006delC	p.P669Qfs^a^4	frameshift	F	Novel mutation
EXT2	2	c.273delT	p.F91Lfs^a^21	frameshift	F	Novel mutation
	2	c.310delA	p.I104Sfs^a^8	frameshift	F	Novel mutation
	5	c.722C>T	p.Q258^a^	nonsense	S	Francannet et al. (2001)
	5	c.817C>T	p.Q273^a^	nonsense	F	Vanita et al. (2009)
	5/IVS5	c.934_939+3del	p.L312_Q313del	frameshift/splicing	F	Novel mutation
	7	c.1110delG^c^	p.M370Ifs^a^66	frameshift	S/F^c^	Novel mutation
	8	c.1177delC	p.R393Gfs^a^43	frameshift	F	Novel mutation
	7–10	exons 7–10 deletion		deletion	S	Novel mutation
	8	exon 8 deletion		deletion	F	Jennes et al. (2009)

^a^The reference sequences for *EXT1* and *EXT2* genes are NM_000127 and NM_207122.1, respectively

Only deleted exons in *EXT2* are numbered according to the reference sequence NM_000401.3, as counted by the manufacturer of “*SALSA MLPA probemix P215*-*B2 EXT*” description version 10 (MRC Holland)

*F* familial

*S* sporadic

^b^mutation detected in two unrelated patients, both familial cases

^c^mutation detected in two unrelated patients, one sporadic and one familial case

In all sporadic cases, presence of the mutation was excluded in both healthy parents, thus confirming their de novo occurrence in the probands. In familial cases, the identified alterations were checked for co-segregation with the phenotype and were not shown in the unaffected family members. Furthermore, all three missense variants were predicted to be probably damaging in most of the in silico analyses performed by us with the use of Mutation Taster 2, PolyPhen2, and SIFT software (Table 3).

**Table 3:** Location of the missense mutations identified in our patients in reference to the EXT1 domain organization. Pathogenicity of each variant was assessed by Mutation Taster 2, Polyphen-2, and SIFT

Case no	Inheritance pattern	Reference to literature	EXT1 mutation at cDNA and protein level	Location at the EXT1 domain level	Mutation Taster 2 prediction	SIFT score	PolyPhen-2 score
1	AD (familial)	Known	c.812A>G (p.Y271C)	exostosin domain	disease causing	0.05 (damaging)	1.000 (probably damaging)
2	AD (familial)	Known	c.1019G>A (p.R340H)	exostosin domain	disease causing	0.39 (tolerated)	0.948 (possibly damaging)
3	AD (familial)	Known	c.1036A>G (p.R346G)	exostosin domain	disease causing	0.02 (damaging)	0.940 (possibly damaging)

*AD* autosomal dominant

MutationTaster 2: MutationTaster employs a Bayes classifier to eventually predict the disease potential of an alteration. The Bayes classifier is fed with the outcome of all tests and the features of the alterations and calculates probabilities for the alteration to be either a *disease mutation* or a harmless *polymorphism*

SIFT (sorting intolerant from tolerant): the amino acid substitution is predicted damaging if the score is <0.05, and tolerated if the score is ≥0.05

PolyPhen-2: score ranges from 0 to 1. The amino acid substitution is predicted damaging if the score is above 0.85

## Discussion

Hereditary multiple exostoses is a relatively frequent autosomal dominant bone disorder resulting from heterozygous inactivating mutations of *EXT1* and *EXT2* genes. Both gene products represent tumor suppressor proteins involved in heparan sulphate (HS) synthesis and cartilage formation. EXT1 and EXT2 act in a hetero-oligomeric complex and catalyze the elongation of HS chains (McCormick et al. 2000; Busse et al. 2007). Mutations in EXT1 or EXT2 are believed to result in reduced level of HS biosynthesis as well as in shortening of HS chains, what disrupts the gradient of morphogens and impair signal transduction in the epiphyseal growth plate in the cartilage. This leads to the loss of cell polarity and once occur in the peripheral part of bone, the cells maintain to proliferate, bring other wild-type cells along and grow into a cartilaginous cap, i.e., osteochondroma (Jones 2011; de Andrea and Hogendoorn 2012).

There are only a few reports on large HME cohorts studied with a comprehensive molecular diagnostic testing including both gene sequencing and quantitative assays, such as MLPA or quantitative PCR (qPCR) for all exons (Porter et al. 2004; Jennes et al. 2009; Ciavarella et al. 2013). Importantly, such studies have never been performed in Polish patients. Thus, our analysis represents the first source of information on molecular cause, frequencies, and the proportion of *EXT1* and *EXT2* mutations in Polish patients affected by HME. In our study, we demonstrated *EXT1* and *EXT2* mutations in 28 (84.9 %) out of 33 unrelated probands, which represents a similar diagnostic success rate to previously published reports in which this value varied from 70 to 95 % (Jennes et al. 2009; Ciavarella et al. 2013). Importantly, in *EXT1*/*EXT2* positive Polish patients, *EXT1* mutations were found almost twice more frequently than *EXT2* mutations (18 vs 10 hits, i.e., 64.3 % vs 35.7 % respectively), which is also in accordance with the literature data (Jennes et al. 2009). The majority of MO causing changes (75–80 %) represent inactivating mutations, i.e., nonsense, frameshift, and splice-site (Jennes et al. 2009). Likewise, as shown in Table 2, 23 out of 28 mutations (82 %) detected in our cohort were also leading to the premature truncation of the protein product. In addition, we found three different *EXT1* missense substitutions associated with HME phenotype: p.Y271C, p.R340H, and p.R346G (Fokkema et al. 2011; Raskind et al. 1998; Signori et al. 2007). All missense substitutions were located within exostosin domain of EXT1 and were predicted to be probably damaging to the protein function in computational analyses by means of PolyPhen2, SIFT, and Mutation Taster2 software. An overview of missense mutations, along with their intragenic location, and predictive values of in silico analyses is presented in Table 3.

In keeping with the past reports, *EXT1* mutations are usually distributed throughout the entire protein sequence, while *EXT2* lesions cluster in the first half of the protein (Jennes et al. 2009). However, in a large Italian cohort described by Ciavarella et al. (2013), 50.1 % of all *EXT1* mutations were localized in exons 1 and 2. Our findings based on Polish patients support the hypothesis that there may be an excess of mutations in the first two exons of *EXT1*. In our cohort, mutation in exons 1 or 2 was identified in ten of 18 (55.6 %) *EXT1* mutation carriers, which comprised 30.3 % of our initial cohort (ten probands out of 33). Therefore, we propose that diagnostic screening of HME Polish patients should start with the sequencing of the first two exons of *EXT1*, followed by sequencing of the rest of the gene. Next, in the case of negative results, we suggest *EXT2* sequencing followed by copy number assays for *EXT1*/*EXT2* exons.

To conclude, our paper is the first report that provides the results of exhaustive mutational screening of both HME related genes (*EXT1*/*EXT2*) in a Polish cohort. The ratio of *EXT1* vs *EXT2* gene lesions in our study is in full accordance with the relative mutational frequencies published previously for other Caucasian cohorts. We also expand here the mutational spectrum associated with MOs by describing the 15 novel pathogenic alterations in the *EXT1* and *EXT2* genes. In addition, although based on a small sample, our study supports the hypothesis that at least in certain groups of patients *EXT1* mutations may cluster within the first two exons of the gene. This important finding should influence the diagnostic algorithm, thereby leading to the optimization of cost-effectiveness rate in HME genetic testing. Furthermore, we showed that intragenic *EXT1*/*2* deletions may account for a non-negligible proportion of HME causative mutations. Therefore, we propose that MLPA or qPCR should be implemented into routine molecular diagnostic of the *EXT1*/*2* genes, especially if the sequence analysis detects no pathogenic alteration.
